# Comparison of variant callers using 60 532 multi-ancestry whole genome sequences

**DOI:** 10.1093/bib/bbag130

**Published:** 2026-03-27

**Authors:** Hufeng Zhou, Zilin Li, Derek Shyr, Xihao Li, Haoyu Yang, Rounak Dey, Yushi Tang, Robert Maier, Eric Boerwinkle, Steve Buyske, Mark Daly, Adam Felsenfeld, Richard A Gibbs, Namrata Gupta, Ira M Hall, Tara Matise, Ginger A Metcalf, Albert Smith, Catherine Reeves, Heidi J Sofia, Nathan O Stitziel, Michael C Zody, NHGRI Genome Sequencing Program (GSP) Consortium, Benjamin Neale, Xihong Lin

**Affiliations:** Department of Biostatistics, Harvard T.H. Chan School of Public Health, 677 Huntington Avenue, Boston, MA 02115, United States; Department of Biostatistics, Harvard T.H. Chan School of Public Health, 677 Huntington Avenue, Boston, MA 02115, United States; Department of Biostatistics, Harvard T.H. Chan School of Public Health, 677 Huntington Avenue, Boston, MA 02115, United States; Department of Biostatistics, University of North Carolina at Chapel Hill, 3101 McGavran-Greenberg Hall, 135 Dauer Drive, Chapel Hill, NC 27599, United States; Department of Genetics, University of North Carolina at Chapel Hill, 120 Mason Farm Road, Chapel Hill, NC 27599, United States; Department of Biostatistics, Harvard T.H. Chan School of Public Health, 677 Huntington Avenue, Boston, MA 02115, United States; Department of Biostatistics, Harvard T.H. Chan School of Public Health, 677 Huntington Avenue, Boston, MA 02115, United States; Lewis-Sigler Institute for Integrative Genomics, Princeton University, Washington Road, Princeton, NJ 08544, USA; Massachusetts General Hospital, Analytic and Translational Genetics Unit, 185 Cambridge Street, Boston, MA 02114, United States; Stanley Center for Psychiatric Research, Broad Institute of MIT and Harvard, 75 Ames Street, Cambridge, MA 02142, United States; School of Public Health, University of Texas Health Science Center at Houston, 1200 Pressler Street, Houston, TX 77030, United States; Department of Statistics, Rutgers, The State University of New Jersey, 110 Frelinghuysen Road, Piscataway, NJ 08854, United States; Massachusetts General Hospital, Analytic and Translational Genetics Unit, 185 Cambridge Street, Boston, MA 02114, United States; Stanley Center for Psychiatric Research, Broad Institute of MIT and Harvard, 75 Ames Street, Cambridge, MA 02142, United States; Program in Medical and Population Genetics, Broad Institute of Harvard and MIT, 415 Main Street, Cambridge, MA 02142, United States; National Human Genome Research Institute, 9000 Rockville Pike, Bethesda, MD 20892, United States; Department of Molecular and Human Genetics, Baylor College of Medicine, One Baylor Plaza, Houston, TX 77030, United States; Massachusetts General Hospital, Analytic and Translational Genetics Unit, 185 Cambridge Street, Boston, MA 02114, United States; Stanley Center for Psychiatric Research, Broad Institute of MIT and Harvard, 75 Ames Street, Cambridge, MA 02142, United States; Center for Genomic Health, Department of Genetics, Yale School of Medicine, 333 Cedar Street, New Haven, CT 06510, United States; Department of Genetics, Rutgers University, 145 Bevier Road, Piscataway, NJ 08854, United States; Department of Molecular and Human Genetics, Baylor College of Medicine, One Baylor Plaza, Houston, TX 77030, United States; Department of Biostatistics, University of Michigan, 1415 Washington Heights, Ann Arbor, MI 48109, United States; New York Genome Center, 101 Avenue of the Americas, New York, NY 10013, United States; National Human Genome Research Institute, 9000 Rockville Pike, Bethesda, MD 20892, United States; Department of Medicine, Washington University School of Medicine, 660 South Euclid Avenue, St. Louis, MO 63110, United States; Department of Genetics, Washington University School of Medicine, 660 South Euclid Avenue, St. Louis, MO 63110, United States; New York Genome Center, 101 Avenue of the Americas, New York, NY 10013, United States; Massachusetts General Hospital, Analytic and Translational Genetics Unit, 185 Cambridge Street, Boston, MA 02114, United States; Stanley Center for Psychiatric Research, Broad Institute of MIT and Harvard, 75 Ames Street, Cambridge, MA 02142, United States; Program in Medical and Population Genetics, Broad Institute of Harvard and MIT, 415 Main Street, Cambridge, MA 02142, United States; Department of Biostatistics, Harvard T.H. Chan School of Public Health, 677 Huntington Avenue, Boston, MA 02115, United States; Program in Medical and Population Genetics, Broad Institute of Harvard and MIT, 415 Main Street, Cambridge, MA 02142, United States; Department of Statistics, Harvard University, 1 Oxford Street, Cambridge, MA 02138, United States

**Keywords:** whole genome sequencing, variant calling, quality control, genetics, comparison

## Abstract

Whole genome sequencing (WGS) studies play a pivotal role in studying the genetic underpinnings of human diseases and traits. High quality and reproducible variant calling is the cornerstone for the success of downstream analyses, including WGS association studies and polygenic risk prediction. This paper compares the data quality, performance, and concordance of two widely used WGS variant callers, the Genome Analysis Toolkit (GATK) and Variant Tool set that discovers short variants (VT), using 60 532 multi-ancestry whole genomes sequenced by the Centers for Common Disease Genomics (CCDGs) of the NHGRI Genome Sequencing Program. Our findings show that both QCed GATK and VT pipelines yield highly consistent and reliable called Single Nucleotide Variants (SNVs) in large-scale WGS studies, supporting their agreements in joint variants calling. However, the two pipelines exhibit greater discrepancies in calling insertions and deletions (INDELs).

## Introduction

Large scale multi-ancestry Whole Genome Sequencing (WGS) studies provide an unprecedented opportunity to study the effects of common and rare variants on diseases and traits. Examples include the Trans-Omics for Precision Medicine (TOPMed) Program [1] of the US National Heart, Lung, and Blood Institute (NHLBI), and the Genome Sequencing Project (GSP) [2] of the US National Human Genome Research Institute’s (NHGRI), the UK Biobank [3], and the All of Us Research Program [4] of the US National Institute of Health.

High-quality and reproducible variant calling plays a pivotal role in identifying genetic variants in WGS studies and ensuring the success of downstream analysis and genetic discoveries. A WGS variant calling pipeline typically involves read alignment, variant calling, and quality control (QC). These large-scale WGS studies employ different variant calling methods for the joint calling of WGS data from multiple sequencing centers. For example, TOPMed uses VT [5] for joint calling, while GSP uses GATK [6, 7] for joint calling. These variant calling pipelines often differ in their approaches to read alignment, variant calling, and QC procedures. Comparing different variant calling methods using the same large WGS dataset is essential to evaluate their accuracy, consistency, and reproducibility in identifying genetic variants in WGS studies, ensuring the validity of subsequent pooled or meta-analysis.

In this paper, we evaluate the agreements and compatibility between the two popular variant calling methods VT [5] and GATK [6, 7] using the same set of data from a large WGS study. Specifically, our study consists of 60 532 multi-ancestry individuals from the GSP Centers for Common Disease Genomics (CCDG) Freeze 2 WGS data, revealing over 400 million common and rare variants. The study cohort comprises ~40% of inferred European ancestry (e.g. European, European American), 24% African ancestry (e.g. African, African American, African Caribbean), 18% Hispanic/Latino ancestry (including Mexican, Central American, South American, Cuban, Dominican, Puerto Rican), 6% Asian ancestry (e.g. East Asian, Southeast Asian, Asian American), and 12% ‘Other’ ancestry (including individuals with mixed ancestry or those unassigned to a major continental group) with multiple complex disease phenotypes, including cardiovascular, immune-mediated, and neuropsychiatric diseases [8]. Extensive variant and sample QC processes specific to the VT and GATK variant calling pipelines were applied to generate the VT and GATK callsets (Fig. S1).

## Materials and methods

The Genome Analysis Toolkit (GATK) and the Variant Tool set that discovers short variants (VT), which are commonly used in large-scale WGS studies, such as the Trans-Omics for Precision Medicine (TOPMed) Program [1] of the US National Heart, Lung, and Blood Institute (NHLBI), and the Genome Sequencing Project (GSP) [2] of the US National Human Genome Research Institute’s (NHGRI), the UK Biobank [3], and the All of Us Research Program [4] of the US National Institute of Health.

### The quality control procedure of the CCDG VT joint callset

The CCDG VT joint callset was generated using the GotCloud VT pipeline [5], with a comprehensive multi-center and multi-stage QC workflow. Reads alignment to the GRCh38 reference genome, followed by joint genotype calling and variant refinement using haplotype-based methods and support vector machine (SVM)-based filtering. Variant discovery was performed on a per-sample, per-chromosome basis, with high-quality read evidence, and normalization was applied accordingly. Biallelic genotype data were produced from merging candidate variants across samples. Joint genotyping was performed hierarchically in batches using genotype likelihoods to reduce spurious heterozygotes. A comprehensive set of variant level and genotype level features was collected, including depth, Hardy–Weinberg equilibrium (HWE), allele balance, and bias metrics. Genotypes that did not meet quality thresholds were set to missing. An SVM was trained on one chromosome and then applied genome-wide and additional filters of excess heterozygosity based on HWE (EXHET) and Mendelian or duplicate inconsistencies (DISC) were applied. All variants after QC were kept for downstream analyses.

Sample-level QC was performed using stringent thresholds to ensure high-confidence genotype calls across the cohort. Samples were excluded with excessively low (<10×) or high (>60×) depth, elevated contamination (FREEMIX >5%), or inadequate genome-wide coverage (FRAC_DP10 < 0.80 or FRAC_DP20 < 0.60).

For comparisons with GATK, both callsets were harmonized by left-alignment, trimming, and decomposition to biallelics using the same GRCh38 reference and contig set, and identical low-complexity and callable-region masks were applied, with HWE and call rate statistics summarized by ancestry. Together, these steps yield a VT callset whose QC strategy parallels conventional call-rate, HWE, QD, and GQ filters but further incorporates pedigree- and duplicate-informed supervised classification to improve specificity, while ensuring comparability with other pipelines through harmonized normalization.

### The quality control procedure of the CCDG GATK joint callset

Using HiSeqX and NovaSeq technologies, the GSP CCDG [2] Freeze 2 callset has an average sequencing depth of 30X. The 60 532 multi-ancestry samples were sequenced in four genome sequencing centers (Baylor College of Medicine, Broad Institute of MIT and Harvard, Washington University at St Louis, and New York Genome Sequencing Center [8]. All the samples were jointly called at the Broad Institute. Initial sequencing data were mapped to a reference genome using BWA, followed by joint variant calling with the GATK pipeline. The jointly called WGS data were then QCed using Hail [9] and python, where sample QC and variant QC were performed by eliminating samples and variants not meeting sequencing quality metrics.

In the GATK variant QC process, low-quality variants were removed based on several criteria, including insufficient sequencing depth, missing allele count (AC), excessively high average depth, location in low-complexity regions (LCRs), failure in variant quality score recalibration (VQSR), low quality by depth (QD < 4), low call rate (CR < 0.98), and deviation from HWE. HWE filtering was applied at the significance threshold of 10^−5^ for common variants (minor allele frequency [MAF] > 1%) and 10^−6^ for rare variants (MAF < 1%). Thresholds for low-complexity regions (LCRs), call rate (CR), and HWE were calculated and applied separately within each ancestry cohort. Variant information was then combining variant information across samples. Genotypes were processed using sequencing quality indicators, with low-quality alleles set to missing. Homozygous variant genotypes were retained only if they showed low likelihood of being homozygous reference (PL[0] < 20).

For the GATK sample QC process, within each estimated ancestry group [8], we calculated the transition/transversion (Ti/Tv) ratio, heterozygous/homozygous (Het/Hom) ratio, and insertion/deletion (Ins/Del) ratio, and identified outlier samples based on these metrics. Samples that deviated by more than six median absolute deviations from the median were classified as outliers and were excluded. These QC steps effectively removed batch effects across sequencing centers and ethnicities, with a rise in the Ti/Tv ratio indicating enhanced variant data quality. Transition/transversion, heterozygous/homozygous, and insertion/deletion ratios were calculated for pass QC variants, and compared between sequencing centers and ancestries. The results show the QC process resulted in a consistent, batch-effect-free dataset (Fig. S1).

### Ancestry estimation of the GSP CCDG Freeze 2

The GSP-CCDG study consists of multi-ancestries, including European, African American, Hispanic, East Asian, and South Asian. Homogeneous ancestry groups were estimated using the two-step semi-supervised method [8]. Our method combines Random Forest and Gaussian Mixtures clustering for refining ethnicity predictions. At the first step, we train a random forest model on the reference 1000 Genome data to predict homogeneous genetic ancestry groups. We randomly selected markers from the 1000 Genomes project, using autosomes and common, linkage disequilibrium-pruned variants, yielding 173 000 variants. We applied the trained model to the CCDF Freeze 2 dataset. This was achieved by using the TRACE method, a modified PCA approach, to project CCDG Freeze 2 samples onto 1000G-based PC space. Since the 1000 Genomes dataset lacks sufficiently diverse admixture populations, ancestrally heterogeneous samples that do not belong to the 1000 Genome populations are classified as ‘other.’ In the second step, we applied the unsupervised model-based clustering method to identify homogeneous genetic ancestry groups within ‘other.’ HWE testing was then conducted within each homogenous ancestry group. There are no trios or duos in CCDG Freeze 2 and no technical replicates available in our analysis cohort; therefore, Mendelian error rates and replicate concordance cannot be computed.

### Variant-level comparison of the GATK and VT callsets

To ensure that the observed discrepancies were not due to technical artifacts, both callsets were fully harmonized by left-alignment, trimming, and decomposition into biallelics using the same GRCh38 reference to ensure precise comparison between these two callsets.

To compare the GATK and VT callsets, we first extracted all quality-controlled variants from each callset (stored in aGDS [10] files) and compared them based on chromosomes, positions, reference alleles, and alternate alleles. If all four fields matched, the variant was considered present in both callsets. Variants found only in one callset were identified as either GATK-only or VT-only variants. Minor allele frequency (MAF) calculations were performed for these variants using data from 60 532 samples, with analyses conducted separately for single nucleotide variants (SNVs) and insertions/deletions (INDELs).

The variant-level comparison assesses the overlap of the called QCed SNVs and INDELs between the GATK and VT callsets. We conducted comparisons using all variants and further stratified them by Minor allele counts (MACs) and MAFs, grouping variants into distinct categories: MAC = 0, singletons, doubletons, doubletons with MAF < 0.1%, variants with MAF between 0.1% and 1%, and variants with MAF > 1% (Tables S1–S3 and Figs S2–S4).

### Genotype-level comparison

The genotype-level comparison evaluates the agreement of individual genotypes between the shared variants of the GATK and VT callsets. We performed a comparison of the GATK genotype matrix and the VT genotype matrix that include all the overlapping variants. Specifically, we extracted the genotype matrices of 403 596 454 overlapping variants across 60 532 samples from both callsets and compared the agreement between the two large 403 596 454 × 60 532 genotype matrices.

We turned the genotype value (e.g. 0/0,0/1,1/1) to dosage value (e.g. 0,1,2) to facilitate comparisons, and evaluated the degree of agreement for each entry between the GATK and VT genotype matrices. We calculated the percentages of the variants that have matched and mismatched genotype values (Tables 1 and 2).

**Table 1:** Overall agreement of the genotype matrices between the GATK and VT callsets for the overlapping PASS variants.

	VT hom ref (0)	VT het [1]	VT hom alt [2]	VT NA
GATK hom ref (0)	98.8%	0.002%	0.0007%	0.016%
GATK het [1]	0.002%	0.54%	0.0006%	0.00008%
GATK hom alt [2]	0.0006%	0.0001%	0.31%	0.0005%
GATK NA	0.30%	0.0003%	0.0007%	0.00003%

Link Text: Genotype concordance matrix between GATK and VT callsets.

**Table 2:** The agreement of genotypes and the disagreement due to missingness between the GATK and VT callsets by minor allele frequency (MAF).

Category	MAF	Agreement	Disagreement Due to Missingness
All		99.67%	97.90%
Common	>0.05	99.60%	55.20%
Low-Frequency	(0.01, 0.05]	99.50%	91.70%
Rare	(1E – 03, 1E – 02]	99.50%	97.70%
Very Rare	(1E – 04, 1E – 03]	99.50%	99.20%
Extremely Rare	<1E – 04	99.70%	99.00%

Link Text: Genotype missingness-related disagreement by MAF category.

### Comparison of single variant GWAS analyses using the two callsets

**Figure 1:**
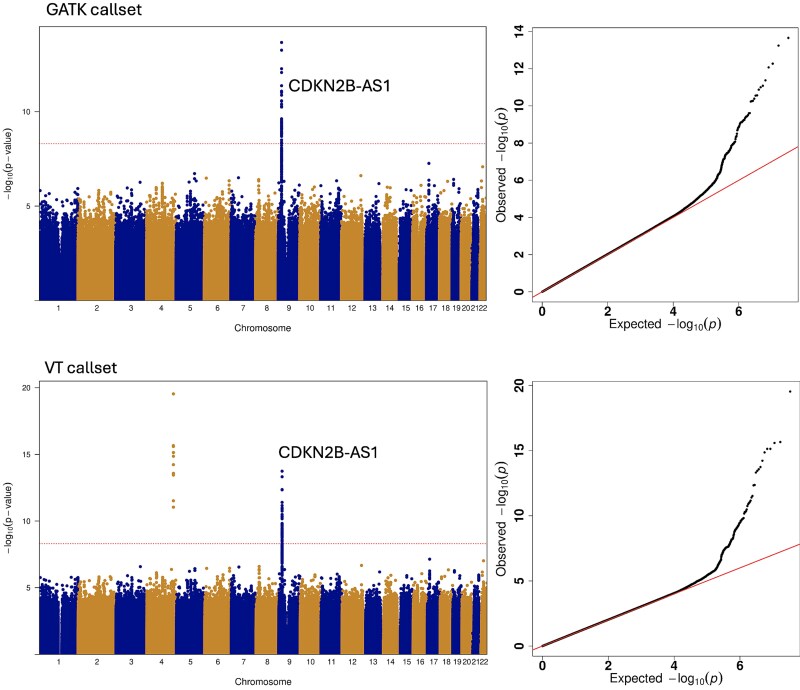
Comparison of the single common variant (MAF > 1%) GWAS analysis results of early onset CVD between the GATK and VT callsets, visualized in Manhattan plots and QQ plots. Shows the GATK and VT callsets are reliable and agree with each other in GWAS analysis.

We compared single-variant association results for early-onset coronary artery disease (EO-CAD) between the GATK and VT callsets. The CCDG Freeze 2 has 7939 CVD cases and 12 229 controls across the multi-ancestry cohort. The GWAS analysis restricted to variants with MAF > 1%. Logistic mixed models using the sparse Generated Related Matrix (GRM) to account for relatedness were fit by adjusting for age, sex, sequencing centers, and 20 ancestry PCs. The results are compared in Fig. 1.

## Results

The VT and GATK variant callsets that were derived from the same large CCDG WGS samples offer a unique opportunity to study the consistency of the called genotypes using the GATK and VT pipelines. We performed a systematic comparison of variant-level, genotype-level and single variant association analyses between the VT and GATK callsets.

### Variant-level comparison of the GATK and VT callsets

There is a total of 578 424 985 called variants (SNVs+INDELs) before QC and 444 107 715 called variants after QC in the GATK callset. Similarly, there are a total of 673 282 100 called variants before QC and 591 688 214 called variants after QC in the VT callset. Among the pass-QC variants of the GATK callset, 2170 variants have minor allele count equal to 0 (MAC = 0), while the pass-QC variants of the VT callset consist of a much larger number of SNVs 144 080 818 SNVs that have MAC = 0 (Table S1). After removing the variants with MAC = 0, the two callsets have a similar number of pass-QC variants (444 105 545 variants for GATK and 447 607 396 variants for VT).

When comparing the variant-level (SNVs+INDELs) agreement between the VT and GATK callsets, 403 596 454 variants were found to be in common between the callsets (Table S1). After excluding the variants with the GATK-only MAC = 0 variants and the VT-only MAC = 0 variants, 83% of the variants are shared between the two callsets (Fig. S2).

We found that the number of shared variants between GATK and VT callsets is significantly higher for SNVs (88%) compared to INDELs (23%) (Tables S2 and S3; Figs S3 and S4). The notably higher agreement for SNVs compared to INDELs across the two pipelines emphasizes the technical challenges and variability inherent in INDEL calling. The representation of INDELs in VCF format becomes complex with multi-allelic sites, particularly where multiple SNVs co-occur, complicating the accurate portrayal of quality metrics.

Most variants in WGS studies are rare, with singletons and doubletons accounting for ~60% of all variants. Common variants, e.g. those with MAF > 1%, made up ~2.7% of the total. The variant distributions are comparable between GATK and VT callers (Table S1). For each MAC/MAF category, there is a good agreement of called variants between the two callsets ranging from 80% to 85% (Fig. S1). Singletons have the lowest variant-level agreement between the GATK and VT callsets compared with the other MAC/MAF groups. This is likely due to the challenge of distinguishing singletons from random sequencing artifacts.

When variants are categorized into SNVs and INDELs (Tables S2 and S3; Figs S3 and S4), INDELs show a significantly lower agreement between GATK and VT (14%–38%) compared to SNVs across all MAF categories (80%–85%). Furthermore, the agreement for INDELs between the two methods improves as MAF increases, with variants at higher MAF categories (>1%) exhibiting notably better consistency (38%) than those at lower frequencies (singletons and doubletons) (14%–18%). With proper normalization (left-alignment, trimming, and decomposition into biallelic representations using the same GRCh38), the low INDEL overlap reflects true methodological differences of the two callers rather than inconsistent variant representation.

### Genotype-level comparison

A comparison of the genotype matrices of the shared pass QC SNVs of the GATK and VT callsets shows a very high concordance (99.6%) of the genotypes between the two callsets (Table 1). The high agreement of called genotypes between the two callers was mainly due to the large number of homozygous reference variants they both identified. This high genotype concordance is also observed in this TOPMed studies [1]. For common variants whose genotypes have discrepancy between the two callsets, 55% were attributed to missing values. In contrast, for low frequency and rare variants whose genotypes have discrepancy, over 90% were due to missing values (Table 2). Common variants have more reliable calls and fewer missing values than uncommon and rare variants. Overall, most differences in genotypes between the GATK and VT callsets stem from the missingness (NA in genotype fields) in the GATK callset, accounting for 93% of the genotype matrix-level disagreement (Table S4). Removing these missing values enhances concordance, shown by the lower disagreement rate (Table S5). The majority of discrepancies occur between homozygous reference and heterozygous calls, while homozygous alternate differences are comparatively rare. This pattern is expected given variant representation differences before normalization and small allele-frequency shifts near the variant calling threshold. Overall, the non-reference concordance remains high.

### Comparison of single variant GWAS analyses using the two callsets

Single variant GWAS analysis of common variants (MAF > 1%) for the binary phenotype early-onset cardiovascular disease (EOCVD) using the GATK and VT callsets yielded highly consistent results (Fig. 1). Both the GATK and VT callsets identified the variants in long noncoding RNA CDKN2B-AS1 on chromosome 9 that are associated with EOCVD, which are supported by the extensive literature [11–14]. Additionally, VT identified the variants in intergenic region between LINC02512 and LINC02382 on chromosome 4 that are associated with CVD. This finding was unsupported by the literature, suggesting this signal on chromosome 4 may be false positive.

## Discussion

Our analysis was limited to small variants (SNVs and INDELs), and we did not evaluate structural variants (SVs) or copy-number variants (CNVs). While both GATK and VT pipelines focus primarily on small variant detection, large-scale joint calling of SVs and CNVs remains technically challenging and methodologically distinct. Future work will be needed to systematically compare SV and CNV calling pipelines across multi-ancestry WGS datasets, which will be essential for establishing comprehensive gold-standard references that include both small and large variant classes.

In addition to the GATK and VT pipelines compared in this study, other joint-calling frameworks such as GLnexus (supporting both DeepVariant and GATK gVCFs) and GraphTyper (recently applied to the UK Biobank WGS data) represent promising alternatives for large-scale variant calling. Evaluating these methods on the CCDG data would provide valuable orthogonal benchmarks and help identify the most efficient pipelines for future studies. However, re-running joint calling with these tools on the full Freeze 2 dataset is cost and computationally prohibitive and logistically challenging within the current scope, given the unavailability of the raw BAM files. It is of future research interest to systematically compare GATK, VT, GLnexus, and GraphTyper across large-scale multi-ancestry WGS datasets.

## Conclusions

In summary, our study compares the performance of two commonly used variant callers, GATK and VT, on a large CCDG WGS dataset. Our results demonstrate a strong agreement in SNV calls between GATK and VT, supporting reliable downstream analyses. However, we observe considerable discrepancy in INDEL calls, highlighting the challenges of INDEL detection and the need for improved algorithms to enhance INDEL calling accuracy in WGS.

**Key Points**

- Large-scale evaluation: We analyzed over 60 000 multi-ancestry whole genome sequences to compare two widely used variant calling pipelines—GATK and VT.
- High SNV concordance: Both pipelines produced highly concordant single nucleotide variant (SNV) calls, supporting their interchangeable use in large-scale WGS studies.
- INDEL discrepancies: Significant differences were observed in insertion and deletion (INDEL) detection, particularly among low-frequency variants.
- Genotype-level consistency: Genotype concordance for shared SNVs was >99%, with most discrepancies driven by missingness rather than true discordant calls.
- Practical guidance: Our results inform pipeline selection and highlight the need for caution when interpreting INDEL-based findings in genomic analyses.

## Supplementary Material

CCDGF2-QC-Callers-Compare-v39-SI-022326_bbag130

## Data Availability

Researchers can apply for access to the CCDG data and more information can be found at https://anvilproject.org/data/consortia/CCDG. About 4% of the samples are unregistered on AnVIL currently and registration for the remaining samples is underway.

## Code availability

The code for the analysis presented in this study can be accessed on Github at https://github.com/zhouhufeng/GATK-VT-comparison.

## Concise biographical

Xihong Lin is Professor of Biostatistics and Statistics at Harvard University, with research spanning statistical genetics, genomics, machine learning, and large-scale health data science. She is affiliated with the Harvard T.H. Chan School of Public Health, the Faculty of Arts and Sciences at Harvard University, and the Broad Institute of MIT and Harvard.
