# Supplementary material for: Comparison of variant callers using 60 532 multi-ancestry whole genome sequences
Source: Brief Bioinform. 2026 Mar 27;27(2):bbag130. doi: 10.1093/bib/bbag130 (PMC13023369; doi:10.1093/bib/bbag130)
Supplement: CCDGF2-QC-Callers-Compare-v39-SI-022326_bbag130 [file ccdgf2-qc-callers-compare-v39-si-022326_bbag130.docx]

**Supporting Information for**

**Comparison of variant callers using 60,532 multi-ancestry whole genome sequences**

Hufeng Zhou^1,^ **^+^**, Zilin Li^1,^ **^+^**, Derek Shyr^1^, Xihao Li^2,3^, Haoyu Yang^1^, Rounak Dey^1^, Yushi Tang^4^, Robert Maier ^5,6^, Eric Boerwinkle^7^, Steve Buyske^8^ ,Mark Daly^5, 6, 9^ , Adam Felsenfeld^10^, Richard A. Gibbs^11^, Namrata Gupta^5,6^, Ira M. Hall^12^,Tara Matise^13^ ,Ginger A. Metcalf^11^, Albert Smith^14^, Catherine Reeves^15^, Heidi J. Sofia^10^, Nathan O. Stitziel^16,17^ ,Michael C. Zody^15^, NHGRI Genome Sequencing Program (GSP) Consortium, Benjamin Neale^5,6,9^, Xihong Lin^1, 9,18,*^

^1^ Department of Biostatistics, Harvard T.H. Chan School of Public Health, Boston, MA 02115, USA,

^2^ Department of Biostatistics, University of North Carolina at Chapel Hill, Chapel Hill, NC, USA,

^3^ Department of Genetics, University of North Carolina at Chapel Hill, Chapel Hill, NC, USA,

^4^ Lewis-Sigler Institute for Integrative Genomics, Princeton University, Princeton, NJ 08544

^5^ Analytic and Translational Genetics Unit, Massachusetts General Hospital, Boston, MA, USA,

^6^ Stanley Center for Psychiatric Research, Broad Institute of MIT and Harvard, Cambridge, MA, USA,

^7^ School of Public Health, University of Texas Health Science Center at Houston, TX, USA,

^8^ Department of Statistics, Rutgers, The State University of New Jersey, Piscataway, NJ, USA,

^9^ Program in Medical and Population Genetics, Broad Institute of Harvard and MIT, Cambridge, MA, USA,

^10^ National Human Genome Research Institute, Bethesda, DC, USA,

^11^ Department of Molecular and Human Genetics, Baylor College of Medicine, Houston, TX, USA,

^12^ Center for Genomic Health, Department of Genetics, Yale School of Medicine, New Haven, CT 06510, USA,

^13^ Department of Genetics, Rutgers University, Piscataway, NJ 08854, USA.

14 Department of Biostatistics, University of Michigan, Ann Arbor, MI 48105, USA

^15^ New York Genome Center, New York, NY, USA,

^16^ Department of Medicine, Washington University School of Medicine, St. Louis, MO 63110, USA,

^17^ Department of Genetics, Washington University School of Medicine, St. Louis, MO 63110, USA,

^18^ Department of Statistics, Harvard University, Cambridge, MA 02138, USA.

*Correspondence should be addressed to Xihong Lin

**+** These Authors contributed equally to this work. The authors wish it to be known that, in their opinion, the first two authors should be regarded as Joint First Authors.

**Email:**  [xlin@hsph.harvard.edu](mailto:xlin@hsph.harvard.edu)

**This PDF file includes:**

Figures S1 to S4

Tables S1 to S5

SI References

*
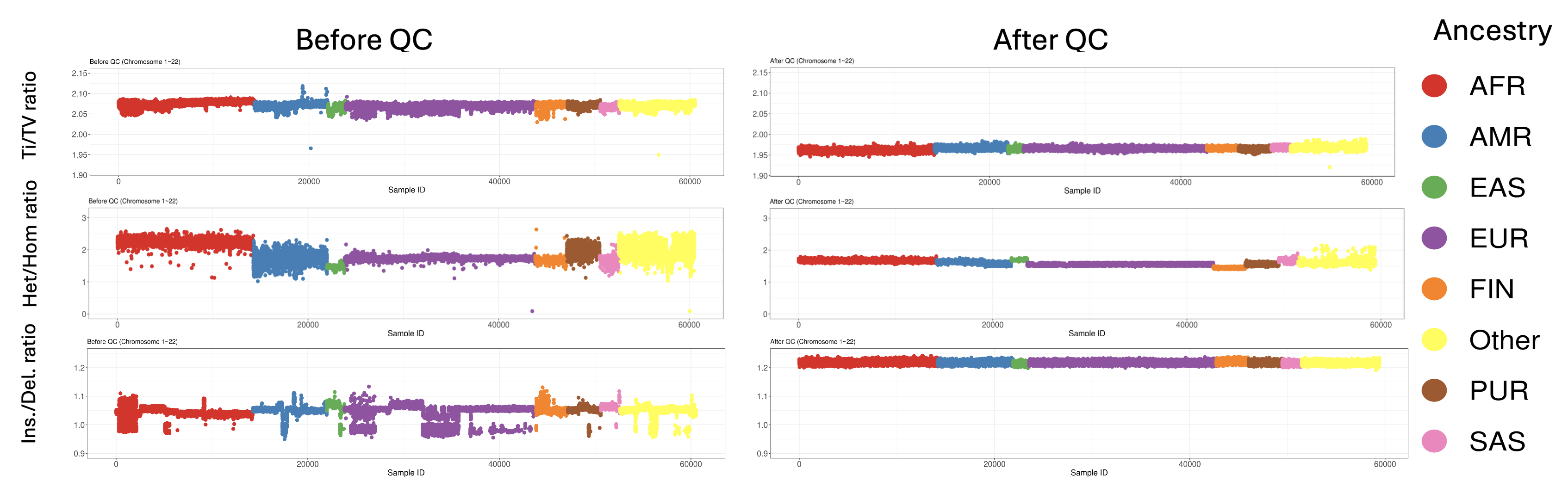
*

**Figure S1.** Ratio plots illustrating improvements in the consistency of the GATK callset before and after quality control (QC). The x-axis represents individual samples, and the y-axes depict the Ti/Tv ratio, Het/Hom ratio, and Insertion/Deletion ratio, respectively. Samples from different ancestries are labeled in distinct colors. The plots show removed batch effects, fewer outliers, enhanced consistency, and overall improved variant quality after QC.


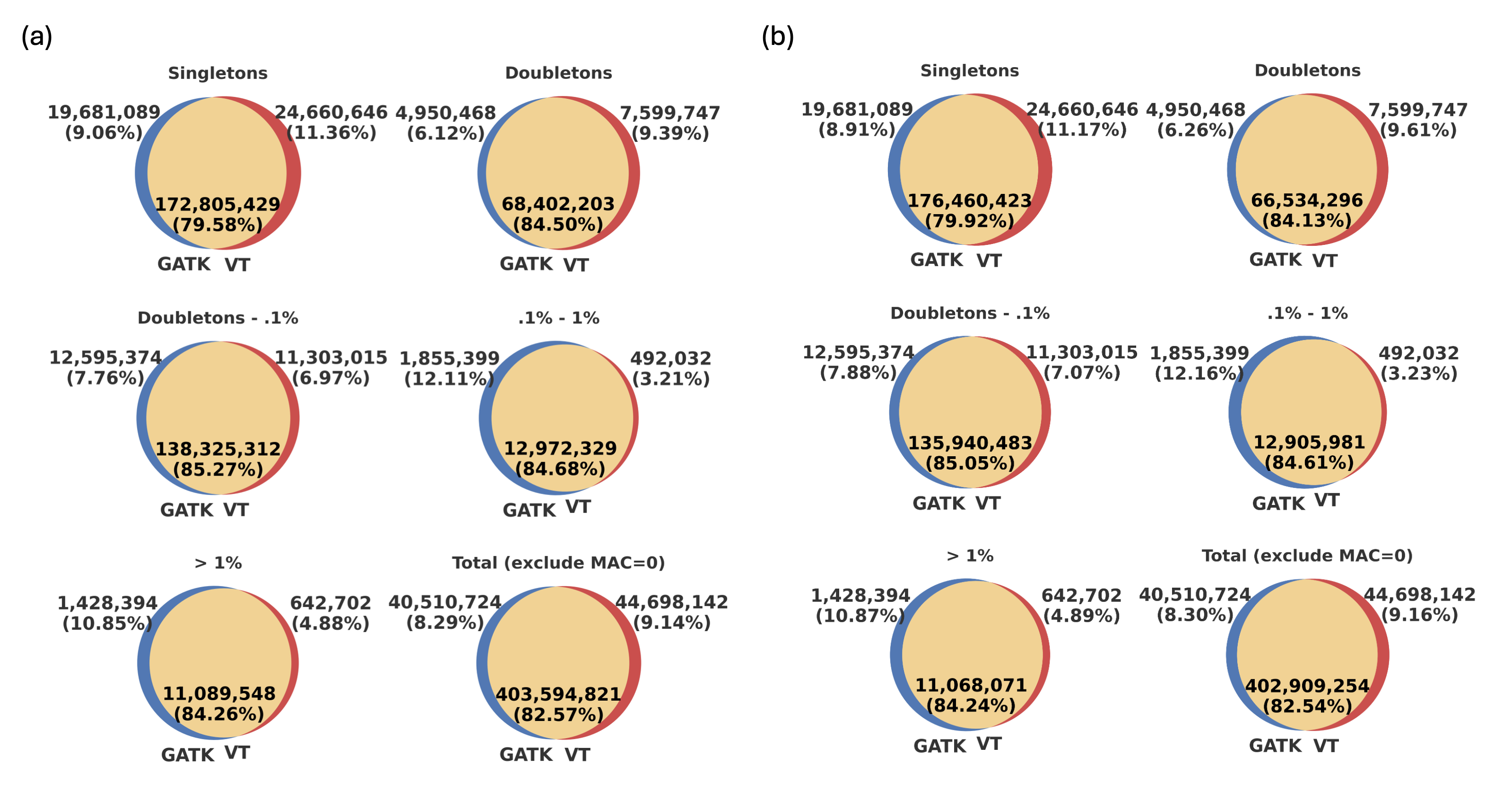


**Figure S2.** Venn Diagrams that show the agreement between the GATK and VT call sets of PASS QC Variants (SNVs+INDELs) in the CCDG Freeze 2 data sets of different Minor Allele Frequency (MAF) groups (Supplementary Table 1). (a) the Venn diagrams generated using the overlapped variants based on the GATK MAFs (2nd column of Supp Table 1). (b) the Venn diagrams generated using the overlapped variants based on the VT MAFs (3rd column of Supp Table 1).


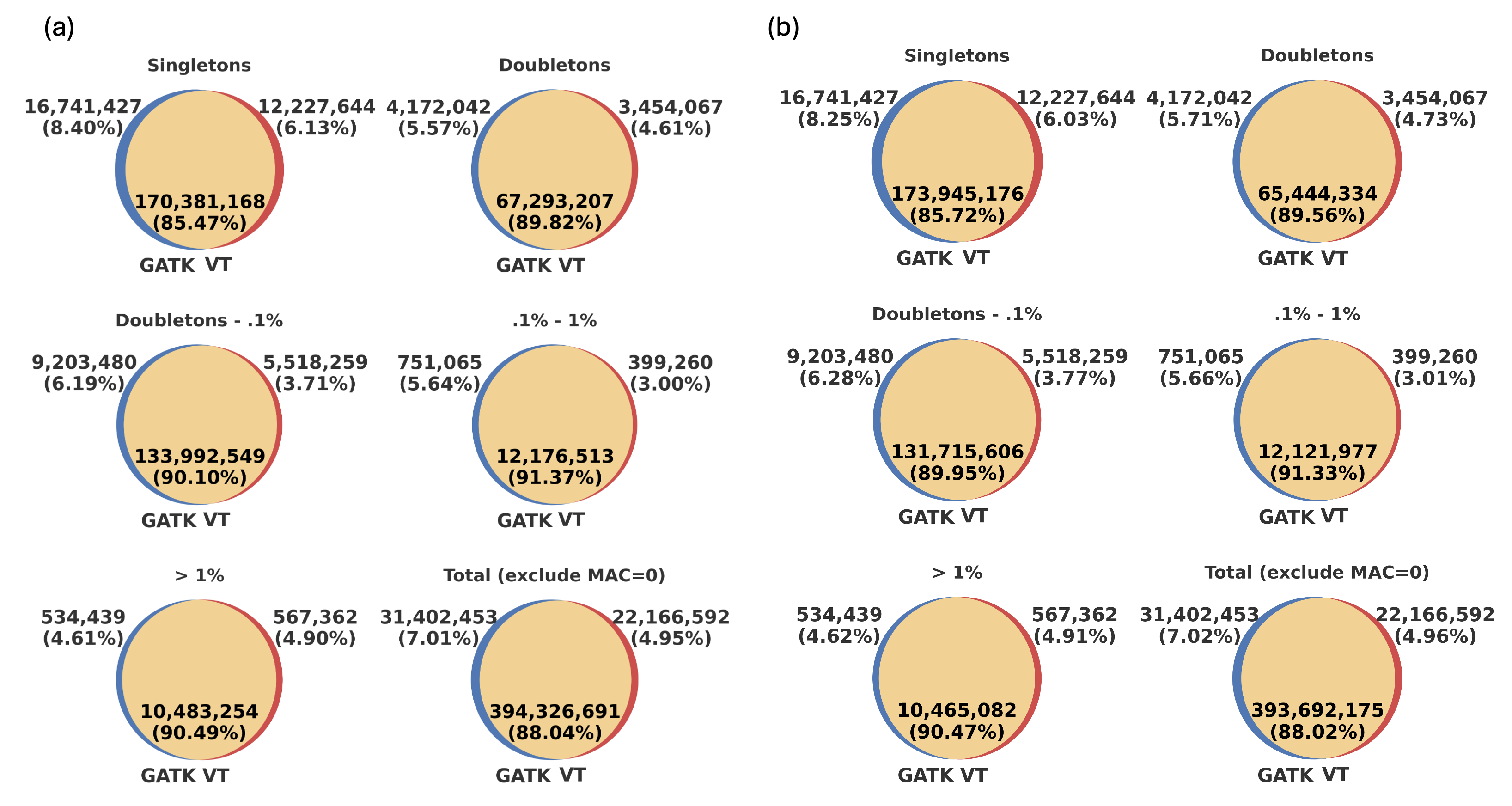


**Figure S3.** Venn Diagrams that show the agreement between the GATK and VT call sets of PASS QC SNVs in the CCDG Freeze 2 data sets of different Minor Allele Frequency (MAF) groups (Supplementary Table 2).

(a) the Venn diagrams generated using the overlapped variants based on the GATK MAFs (2nd column of Supp Table 2). (b) the Venn diagrams generated using the overlap variants based on the VT MAFs (3rd column of Supp Table 2).


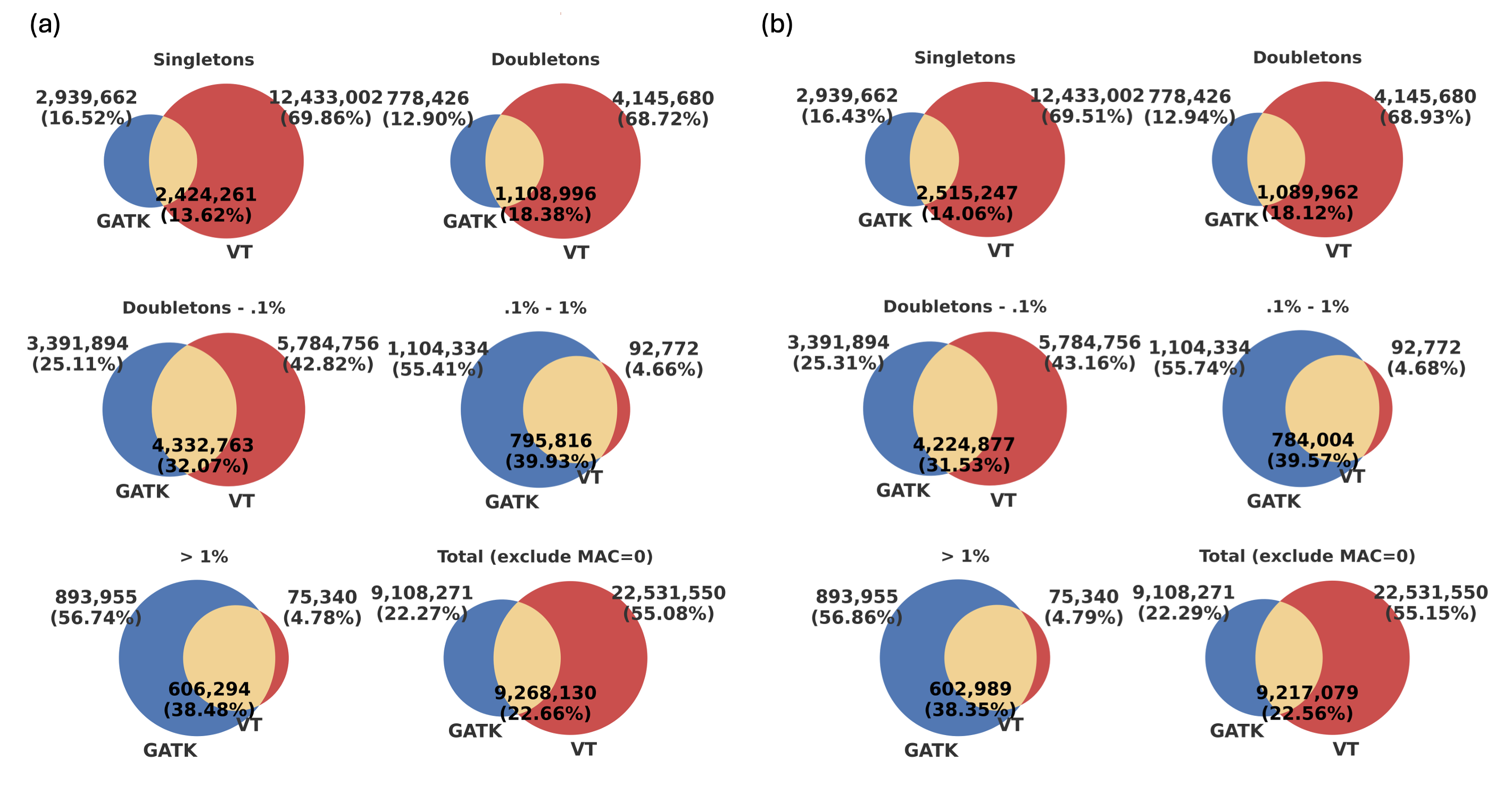


**Figure S4.** Venn Diagrams that show the agreement between the GATK and VT call sets of PASS QC INDELs in the CCDG Freeze 2 data sets of different Minor Allele Frequency (MAF) groups using the GATK MAF-based counts of overlapping INDELs (Supplementary Table 3). (a) the Venn diagrams generated using the overlapped variants based on the GATK MAFs (2nd column of Supp Table 3). (b) the Venn diagrams generated using the overlapped variants of the VT MAFs (3rd column of Supp Table 3).

Table S1. Comparison of the agreement of the variants (SNVs+Indels) that passed QC between the GATK and VT call sets by different Minor Allele Frequency (MAF) groups using the CCDG Freeze 2 callsets.

| Frequency | GATK and VT Overlapped PASS Variants (GATK MAF) | GATK and VT Overlapped PASS Variants  (VT MAF) | GATK Only PASS Variant | VT Only PASS Variant |
| --- | --- | --- | --- | --- |
| MAC=0 | 1,633  (0%) | 687,200  (0.2%) | 537  (0.1%) | 143,393,618 (76.2%) |
| Singletons | 172,805,429 (42.8%) | 176,460,423 (43.7%) | 19,681,089 (48.6%) | 24,660,646 (13.1%) |
| Doubletons | 68,402,203 (16.9%) | 66,534,296 (16.5%) | 4,950,468 (12.2%) | 7,599,747 (4.0%) |
| Doubletons - .1% | 138,325,312 (34.3%) | 135,940,483 (33.7%) | 12,595,374 (31.1%) | 11,303,015 (6.0%) |
| .1% - 1% | 12,972,329 (3.2%) | 12,905,981 (3.2%) | 1,855,399 (4.6%) | 492,032  (0.3%) |
| > 1% | 11,089,548 (2.7%) | 11,068,071 (2.7%) | 1,428,394 (3.5%) | 642,702  (0.3%) |
| Total (exclude MAC=0)* | 403,594,821 | 402,909,254 | 40,510,724 | 44,698,142 |
| Total | 403,596,454 | 403,596,454 | 40,511,261 | 188,091,760 |

*For each column, the count is calculated by subtracting the first row (MAC=0) from the last row (Total).

Table S2. Comparison of the agreement of Single Nucleotide Variants (SNVs) that passed QC between the GATK and VT call sets by different Minor Allele Frequency (MAF) groups using the CCDG Freeze 2 callsets.

| Frequency | GATK and VT Overlapped PASS SNVs (GATK MAF) | GATK and VT Overlapped PASS SNVs (VT MAF) | GATK Only PASS SNVs | VT Only PASS SNVs |
| --- | --- | --- | --- | --- |
| MAC=0 | 1,235  (0%) | 635,751 (0.2%) | 107  (0%) | 132,475,643 (71.4%) |
| Singletons | 170,381,168 (43.1%) | 173,945,176 (44.1%) | 16,741,427 (53.3%) | 12,227,644 (15.0%) |
| Doubletons | 67,293,207 (17.1%) | 65,444,334 (16.6%) | 4,172,042 (13.3%) | 3,454,067 (4.8%) |
| Doubletons - .1% | 133,992,549 (34.0%) | 131,715,606 (33.4%) | 9,203,480 (29.3%) | 5,518,259 (7.8%) |
| .1% - 1% | 12,176,513 (3.1%) | 12,121,977 (3.1%) | 751,065 (2.4%) | 399,260 (0.5%) |
| > 1% | 10,483,254 (2.7%) | 10,465,082 (2.7%) | 534,439 (1.7%) | 567,362 (0.5%) |
| Total (Exclude MAC=0)* | 394,326,691 | 393,692,175 | 31,402,453 | 22,166,592 |
| Total | **394,327,926** | **394,327,926** | **31,402,560** | **154,642,235** |

*For each column, the count is calculated by subtracting the first row (MAC=0) from the last row (Total).

Table S3. Comparison of the agreement of Insertions and Deletions (INDELs) that passed QC between GATK and VT call sets by different Minor Allele Frequency (MAF) groups using the CCDG Freeze 2 callsets.

| Frequency | GATK and VT Overlapped PASS INDELs (GATK MAF) | GATK and VT Overlapped PASS INDELs (VT MAF) | GATK Only PASS INDELs | VT Only PASS INDELs |
| --- | --- | --- | --- | --- |
| MAC=0 | 398  (0.4%) | 51,449  (0.6%) | 430  (0%) | 10,917,975 (32.6%) |
| Singletons | 2,424,261 (26.2%) | 2,515,247 (27.1%) | 2,939,662 (32.3%) | 12,433,002 (37.2%) |
| Doubletons | 1,108,996 (12.0%) | 1,089,962 (11.8%) | 778,426 (8.5%) | 4,145,680 (12.4%) |
| Doubletons - .1% | 4,332,763 (46.7%) | 4,224,877 (45.6%) | 3,391,894 (37.2%) | 5,784,756 (17.3%) |
| .1% - 1% | 795,816  (8.6%) | 784,004 (8.5%) | 1,104,334 (12.1%) | 92,772 (0.3%) |
| > 1% | 606,294 (6.5%) | 602,989 (6.5%) | 893,955 (9.8%) | 75,340 (0.2%) |
| Total (Exclude MAC=0)* | 9,268,130 | 9,217,079 | 9,108,271 | 22,531,550 |
| Total | 9,268,528 | 9,268,528 | 9,108,701 | 33,449,525 |

*For each column, the count is calculated by subtracting the first row (MAC=0) from the last row (Total).

Table S4. The distribution of the genotype discrepancies of the the shared PASS QC variants between the GATK and VT callsets in the CCDG Freeze 2 callsets

|  | VT  hom ref (0) | VT  het (1) | VT  hom alt (2) | VT  NA |
| --- | --- | --- | --- | --- |
| GATK  hom ref (0) | \ | 0.73% | 0.22% | 4.90% |
| GATK  het (1) | 0.75% | \ | 0.29% | 0.03% |
| GATK  hom alt (2) | 0.18% | 0.04% | \ | 0.02% |
| GATK  NA | 92.8% | 0.09% | 0.02% | \ |

Table S5. The distribution of genotype discrepancies between the shared PASS QC variants of the GATK and VT callsets after excluding missing values in the CCDG Freeze 2 callsets

|  | VT  hom ref (0) | VT  het (1) | VT  hom alt (2) |
| --- | --- | --- | --- |
| GATK  hom ref (0) | \ | 34.9% | 10.6% |
| GATK  het (1) | 35.6% | \ | 8.1% |
| GATK  hom alt (2) | 8.7% | 2.1% | \ |

**SI References**

1. K. A. Tryka *et al.*, NCBI's Database of Genotypes and Phenotypes: dbGaP. *Nucleic Acids Res* **42**, D975-979 (2014).

2. M. D. Mailman *et al.*, The NCBI dbGaP database of genotypes and phenotypes. *Nat Genet* **39**, 1181-1186 (2007).
